# A systematic review of health economic models of opioid agonist therapies in maintenance treatment of non-prescription opioid dependence

**DOI:** 10.1186/s13722-017-0071-3

**Published:** 2017-02-24

**Authors:** Mersha Chetty, James J. Kenworthy, Sue Langham, Andrew Walker, William C. N. Dunlop

**Affiliations:** 1 PHMR Ltd, London, UK; 2 Mundipharma International Ltd, Cambridge Science Park, Milton Road, Cambridge, CB4 0GW UK; 3 University of Glasgow, Glasgow, UK

**Keywords:** Systematic review, Non-prescription opioid dependence, Opioid agonist maintenance therapy, Economic evaluation

## Abstract

**Background:**

Opioid dependence is a chronic condition with substantial health, economic and social costs. The study objective was to conduct a systematic review of published health-economic models of opioid agonist therapy for non-prescription opioid dependence, to review the different modelling approaches identified, and to inform future modelling studies.

**Methods:**

Literature searches were conducted in March 2015 in eight electronic databases, supplemented by hand-searching reference lists and searches on six National Health Technology Assessment Agency websites. Studies were included if they: investigated populations that were dependent on non-prescription opioids and were receiving opioid agonist or maintenance therapy; compared any pharmacological maintenance intervention with any other maintenance regimen (including placebo or no treatment); and were health-economic models of any type.

**Results:**

A total of 18 unique models were included. These used a range of modelling approaches, including Markov models (n = 4), decision tree with Monte Carlo simulations (n = 3), decision analysis (n = 3), dynamic transmission models (n = 3), decision tree (n = 1), cohort simulation (n = 1), Bayesian (n = 1), and Monte Carlo simulations (n = 2). Time horizons ranged from 6 months to lifetime. The most common evaluation was cost-utility analysis reporting cost per quality-adjusted life-year (n = 11), followed by cost-effectiveness analysis (n = 4), budget-impact analysis/cost comparison (n = 2) and cost-benefit analysis (n = 1). Most studies took the healthcare provider’s perspective. Only a few models included some wider societal costs, such as productivity loss or costs of drug-related crime, disorder and antisocial behaviour. Costs to individuals and impacts on family and social networks were not included in any model.

**Conclusion:**

A relatively small number of studies of varying quality were found. Strengths and weaknesses relating to model structure, inputs and approach were identified across all the studies. There was no indication of a single standard emerging as a preferred approach. Most studies omitted societal costs, an important issue since the implications of drug abuse extend widely beyond healthcare services. Nevertheless, elements from previous models could together form a framework for future economic evaluations in opioid agonist therapy including all relevant costs and outcomes. This could more adequately support decision-making and policy development for treatment of non-prescription opioid dependence.

**Electronic supplementary material:**

The online version of this article (doi:10.1186/s13722-017-0071-3) contains supplementary material, which is available to authorized users.

## Background

Physical and psychological dependence can occur with any opioid drug, but the non-prescription or ‘street’ use of heroin presents the greatest problems to society [1]. In 2010, the global prevalence of opioid use was estimated at 0.6–0.8% of the population aged 15–64 years (between 26.4 and 36.0 million opioid users), of which approximately half, or between 13 and 21 million, were using heroin [2].

Illicit non-prescription opioid dependence is associated with major medical, personal and social problems, including increased risk of infection with human immunodeficiency virus (HIV) or hepatitis C virus (HCV), increased risk of death due to suicide, overdose or violence, decreased quality of life, high rates of psychiatric co-morbidity, and involvement in criminal activity [3, 4].

It was estimated in 2008 that there are approximately 16 million injecting drug users (IDU) worldwide and that 3 million (18.9%) of them were living with HIV. Global prevalence of HCV infection among injecting drug users in 2010 was 46.7%, meaning that some 7.4 million injecting drug users worldwide are infected with HCV and 2.3 million injecting drug users are infected with hepatitis B [2].

In 2010, illicit drug use was found to be associated with between 99,000 and 253,000 deaths globally, with drug-related deaths accounting for between 0.5 and 1.3% of all-cause mortality among those aged 15–64 years [2].

Heroin addiction also has significant economic costs to society, resulting from the association between crime and opioid dependence. Many opioid-dependent individuals become involved in crime to support their drug use, but crime may also provide the money and the contacts to buy drugs [1]. Based on a review, the average heroin user is likely to engage in criminal activity for 40–60% of the time they are not incarcerated or not in treatment [5].

Other societal impacts result from the psychopharmacological effects of the drug, which may result in mistakes at work, lost productivity or unemployment [6]. Personal relationships may suffer or parental capacity may be hampered. Evidence from the United Kingdom (UK) shows neglect among children is correlated strongly with parental heroin use, and in the United States of America (US) parental problem drug use is one of the commonest reasons for children entering the care system [7].

The economic and social costs of Class A drug use were estimated to be £15.4 billion in 2003–2004 in England and Wales [6], with opioid and/or crack use accounting for 99%. Health and social care costs accounted for £557 million, implying that the majority of the costs are borne outside of health and social care provision.

Effective treatment for opioid dependence is available but is likely to be long-term or even life-long. Options include psychosocial/behavioural assistance, and pharmacological interventions including opioid agonist therapy. The most commonly used medications for opioid substitution include the opioid agonist methadone, and buprenorphine (with/without naloxone), a partial agonist/antagonist combination; less commonly used treatments include naltrexone, morphine sulphate, naloxone, diamorphine, and medical use of heroin [8].

Recent Cochrane reviews, as well as additional studies of maintenance treatment options, have consistently found opioid agonist therapy to be clinically effective and more cost-effective than no drug therapy in opiate-dependent users [1], and have found no major differences in rates of mortality or illicit drug use achieved with these treatments [9].

In financially constrained health systems with finite resources, increasing emphasis is being placed on the ability to demonstrate that healthcare interventions are not only effective, but also cost-effective. Economic evaluations, which use modelling techniques to consider the comparative clinical effects, patient values and cost of care of alternative options, are used by payers and healthcare policymakers to inform the decision-making process.

The emphasis when conducting an economic evaluation is on including all relevant evidence on costs and outcomes. Given the significant impacts on society, the economic framework in economic evaluations of non-prescription opioid dependence should include not only the direct medical costs associated with treatment and preventive interventions, but also costs borne by other areas of society such as social welfare services and the criminal justice system, and indirect costs associated with lost productivity [4].

In order to explore the ways in which previous modelling studies have approached this issue, we undertook a systematic review of published model-based economic evaluations of opioid agonist therapy in treating opioid dependence, including modelling of long-term costs and outcomes and budget impact modelling. The aim was to identify any ‘best practice’ methods for modelling approaches and the costs and outcomes considered, which could be used to guide and inform future health economic models in this area. To our knowledge, this is the first systematic review to examine the modelling approaches that have been applied in opioid agonist therapy for non-prescription opioid dependence.

## Methods

### Search strategy

Search strategies and searches of published literature were designed and performed by an experienced medical librarian. The searches were conducted on 17–18 March 2015 in eight electronic databases: Medline (OvidSP); Medline In-Process Citations and Daily Updates (OvidSP); Embase (OvidSP); the Cochrane Library (Wiley); the Cochrane Database of Systematic Reviews (CDSR) (Wiley); the Database of Abstracts of Reviews of Effects (DARE) (Wiley); the Health Technology Assessment (HTA) Database (Wiley); and the National Health Service Economic Evaluation Database (NHS EED) (Wiley). The databases were searched from the beginning of the database to the date of the search. No date or language limits were applied.

Search terms appropriate for each database were used for the disorder (non-prescription opioid dependence), interventions (any pharmacological maintenance regimen) and study type (health economic studies of any type). The detailed search strategies are presented in Additional file 1.

These searches were supplemented by hand searching of the reference lists of review papers.

In addition, general title searches were conducted, also on 17–18 March 2015, on the websites of HTA agencies in six countries. Health technology assessment (HTA) involves the systematic evaluation of properties, effects and/or impacts of health technologies and interventions. It covers both the direct, intended consequences of technologies and interventions as well as their indirect, unintended consequences, and the HTA approach is used to inform policy and decision-making in health care, especially on how best to allocate limited funds to health interventions and technologies. The assessment is conducted by interdisciplinary groups using explicit analytical frameworks, drawing on clinical, epidemiological, health economic and other information and methodologies. The six HTA agencies selected below are well established and with clearly defined processes and references cases for economic models and were therefore were included in this search.

- Australia: Pharmaceutical Benefits Advisory Committee (PBAC);
- Canada: Canadian Agency for Drugs and Technologies in Health (CADTH);
- England and Wales: National Institute for Health and Clinical Excellence (NICE);
- Germany: Gemeinsamer Bundesausschuss (G-BA); Institute for Quality and Efficiency in Heath Care (IQWIG);
- Scotland: Scottish Medicines Consortium (SMC);
- Sweden: Tandvards-och lakemedelsformansverket (TLV); Swedish Council on Health Technology Assessment (SBU).

These additional searches were intended to identify any additional published or unpublished material missed by the electronic database searches.

### Inclusion and exclusion criteria

The inclusion and exclusion criteria used to select studies for the review are shown in Table 1. Each publication had to fulfil all the inclusion criteria and none of the exclusion criteria to be selected for inclusion in the review. Unless specifically stated otherwise, it was assumed that drug abuse pertained to illicit use of opioids.

**Table 1:** Inclusion and exclusion criteria

Parameter	Criteria
Population	People who are dependent on non-prescription opioids and who are receiving opioid agonist therapy or maintenance therapy for opioid dependency
Intervention	Pharmacological maintenance therapy, monotherapy or combination Morphine/morphine sulphate/diacetylmorphine/diamorphine (DIA) Buprenorphine (BUP) Methadone (METH) Codeine, dihydrocodeine Naloxone, naltrexone (NAL) Buprenorphine/naloxone (BUP/NAL) Note naloxone may be used in combination with other treatments (morphine + naloxone) The following operational definition will be employed for “maintenance” treatment: the treatment approach does not include a reduction or cessation of one of the above treatments as part of the approach
Comparators	Any comparator regime used in maintenance therapy (including no therapy or placebo)
Outcomes	Health economic models (any type including Markov, dynamic, Monte-Carlo, simulations, decision-trees etc)
Study types	Cost-effectiveness (CEA), cost-utility (CUA), cost-minimisation (CMA), cost-benefit (CBA), budget impact (BIM), cost-consequence (CC)
Language	English language abstracts
Timeframe	Last 20 years (1995–2015)
Exclusions	Studies indexed as case reports, case series, editorials and letters English language title and abstracts only Economic studies that do not employ modelling techniques (studies describing extrapolation of data beyond the primary clinical evidence time horizon were considered to include modelling techniques. Studies based only on cost and outcomes during the course of a trial were excluded)

### Study selection

Citations identified by the searches were initially screened for eligibility against the inclusion and exclusion criteria using the title and (where present) the abstract and keywords. Each citation was classified as ‘include’, ‘exclude’ or ‘unsure’. Full text copies were obtained for publications categorised as ‘include’ or ‘unsure’ at the initial screen.

The full-text publications were then screened against the inclusion and exclusion criteria by two independent researchers. Any disagreements were resolved by discussion and consensus. Publications that met all the inclusion criteria and none of the exclusion criteria after full-text review were selected for inclusion in the review. The reason for exclusion was recorded for all studies excluded after full-text review. Excluded publications are listed in Additional file 2.

Information on the modelling approach, perspective, time horizon, comparators and form of evaluation was extracted from each of the included studies and tabulated. Additionally, data were extracted on the type of model used and the range of inputs for costs and outcomes. Data extracted on cost inputs were aimed at addressing the question of what types of costs were used i.e. direct or wider societal and indirect costs, the evidence sources for these types of costs and where gaps may exist in the evidence sources. Data on utility weights for health states in cost-utility analyses were aimed at understanding what values were available, what methods had been used and what gaps might exist.

### Economic appraisal checklist

The model-based economic evaluations were assessed using an adapted form of the checklist for economic evaluations developed by the University of Glasgow [10]. The checklist consists of twelve questions in total, of which nine relate to the economic evaluation itself while the remaining questions address the applicability of results to the local population. Therefore, only the nine questions relating to the quality of the economic evaluation were used in the present analysis. Each of the included studies was graded on each of the questions as ‘yes’, ‘no’ or ‘can’t tell’ by one researcher, and the results tabulated.

## Results

### Search results and study selection

A total of 2666 citations were identified in the electronic literature searches, which decreased to 2149 after removal of duplicates. Hand searching of reference lists and review of HTA websites retrieved a further 14 citations, making a total of 2163 citations. After initial screening, 63 of these progressed to full-text review.

After review of the full text, 45 publications were excluded for the following reasons: non-model or trial-based analysis (n = 18); non-model-based cost analysis (n = 12); review articles (n = 10); HTA reports that did not contain new information of relevance to the review (n = 3); and outdated references, defined as pre-dating 1995 (n = 2). The reference lists of the review articles were hand searched for new references. Three of the HTA reports cited models that had already been captured by the searches and selection process. The outdated references were considered too old to be relevant or useful compared with current treatment programmes. This was in line with the HTA publication by Connock et al. [1], which excluded the same references for the same reason.

**Fig. 1:**
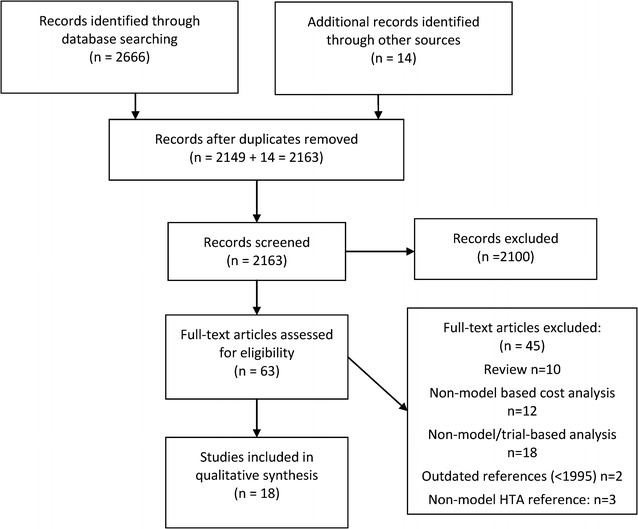
PRISMA flow diagram

A total of 18 unique models were included in the review. Figure 1 shows a preferred reporting items for systematic reviews and meta-analyses (PRISMA) flow diagram for the study screening and selection process.

### Summary of included studies

Twelve of the included models were reported in full-text publications [11–22]. Four were HTA evaluations [1, 23–25]. Of the four HTA reports, two were economic models supporting NICE technology appraisal for naloxone, methadone and buprenorphine in maintenance treatment of opioid dependence, one was the Schering-Plough manufacturer’s submission to the NICE technology assessment (cited in Connock et al. [1]), and one was the SMC advice document based on the manufacturer’s submission by Schering-Plough for suboxone. The remaining two models were published only as abstracts [26–28].

Table 2 summarises the characteristics of the 18 included models.

**Table 2:** Summary of characteristics of included studies

Study/references	Cost year/currency	Country	Form of the evaluation	Perspective taken	Treatments evaluated	Model population	Time horizon	Study design^a^	Outcome measure	Societal costs	Health states
*Journal*-*based publications*											
Barnett [11]	1996 (US $)	US	CEA	US Healthcare provider	METH versus Drug-free treatment	Hypothetical cohort of 1000 25 year old heroin users	Life-time	Markov	Cost/LYG	No	NR
Barnett [12]	1998 (US $)	US	CUA	US Healthcare provider	BMT versus MMT	Hypothetical cohort	10 years	Dynamic model	QALY	No	9 states based on HIV status (uninfected, asymptomatic HIV +ve, AIDS) and drug user status (IDU not on tx, IDU on tx, non-user)
Masson [13]	NR (US $)	US	CEA	US Healthcare provider	MMT versus Enriched Detox	Based on 179 patients in a RCT	10 years	Markov	LYG (base case) QALY (SA)	No	Alive and dead
Negrin [15]	NR/(Euro (€))	Spain	CEA	Drug Treatment centres	3 MMT programmes (high, medium, low intensity)	Based on 586 patients in drug tx centre	1 year	Bayesian	CEAC & CEAPF	NR	NR
Schackman [16]	2010 (US $)	US	CUA	Societal	Office-based BUP/NAL versus no treatment	Hypothetical cohort of stable patients on treatment for 6 months	24 months	Cohort simulation	Cost/QALY	Patient costs	In tx off drugs, Off tx off drugs, In tx on drugs, Off tx on drugs
Sheerin [17]	1999/2000 (NZ $)	New Zealand	CEA	New Zealand Healthcare	MMT	Hypothetical cohort of 1000 IDU	Lifetime	Markov	Cost/LYS	No	HCV + ve, no HCV, Chronic HCV, HCC, Compensated LC, Decompensated LC, Liver transplant, Death
Stephen [18]	2011 (US $)	US	CUA	Societal	MMT versus theoretical course of Deep Brain stimulation	NR	6 months	Decision analytical	QALY	Yes (productivity losses, crime costs)	NA (decision tree)
Tran [19]	2009 (US $)	Vietnam	CUA	Vietnamese Health Service	MMT versus non-MMT	Based on 370 drug users from a cohort study	1 year (5% discounting)	Decision tree	Case of HIV averted QALY of MMT versus non-MMT	No	NA (decision tree)
Zaric [20]	1998 (US $)	US	CUA	US Healthcare provider	Expanding MMT programme (HIV prevalence rate of 5% & 40% versus 15% baseline)	Hypothetical cohort	10 years	Dynamic model	Cost/QALY & cost/LYG	No	10 states based on HIV status (uninfected, asymptomatic HIV +ve, AIDS) and drug user status (IDU not on tx, IDU on tx, non-user) and AID death
Zaric [21]	1998 (US $)	US	CUA	US Healthcare provider	Expanding MMT programme (HIV prevalence rates of 5,10,20, 40%)	Hypothetical cohort	10 years	Dynamic model	QALY and LYG	No	10 states based on HIV status (uninfected, asymptomatic HIV +ve, AIDS) and drug user status (IDU not on tx, IDU on tx, non-user) and AID death
Zarkin [22]	2001 (US $)	US	CBA	Societal	METH	Hypothetical cohort of 1 million adult patients	Lifetime	Monte Carlo simulation model	Cost/benefit ratio	Yes (productivity losses, crime costs)	Heroin non user & not in tx, Heroin user and not in tx, In tx, Incarcerated heroin user, Incarcerated non-user
Miller [14]	NR/(Canadian $)	Canada	Cost Comparison	Societal	MHPP versus non-MHPP	≥20 years old with > 5 year history of injecting heroin, to inject heroin at least daily, and to have previously failed MMT	5 years	Monte Carlo simulation model	Total cost over 5 years	Yes (criminal activity costs)	NA
*HTA*-*sourced models*											
Adi [23]	2004 (GBP £)	UK	CUA	NHS & Societal	NTX versus standard psychosocial care	Hypothetical cohort	1 year	Decision tree with Monte Carlo simulations	QALY	Yes, in a secondary analysis	NA (decision tree)
Connock [1]	2004 (GBP £)	UK	CUA	NHS & Societal	MMT versus BMT versus Placebo	Hypothetical cohort	1 year	Decision tree with Monte Carlo simulations	QALY	Yes, in a secondary analysis	NA (decision tree)
Schering-Plough [24]	2004 (GBP £)	UK	CUA	NHS & PSS	Maintenance versus no drug tx, BUP versus no tx, BUP versus METH	NR	1 year`	Decision tree with Monte Carlo simulations	QALY	NR	NA (decision tree)
SMC [25]	NR/(GBP £)	UK	CUA	NHS & Societal	BUP/NAL versus METH, BUP or no treatment	NR	1 year	Decision analytical	QALY	NR	NR
*Abstracts only*											
Clay [26, 27]	NR/(US $)	US	BIM	US Healthcare provider	BUP/NAL film versus BUP/NAL tablets	Patients initiating treatment for opioid dependence	5 years	Markov model	Cost impact comparing 100% on BUP/NAL film versus 100% on BUP/NAL	No	NR
Fowler [28]	NR/US ($)	US	CUA	NR	MMT versus BMT	Hypothetical cohort of opioid-dependent pregnant women	NR	Decision analytical model	QALY	NR	NR

*AIDS* acquired immunodeficiency syndrome, *BIM* budget impact model, *BMT* buprenorphine maintenance treatment, *BUP* buprenorphine, *BUP/NAL* buprenorphine-naloxone combination, *CBA* cost-benefit analysis, *CEA* cost effectiveness analysis, *CEAC* cost-effectiveness acceptability curve, *CEAPF* cost-effectiveness frontier, *CUA* cost utility analysis, *HCC* Hepatocellular carcinoma, *HCV* hepatitis C virus, *HIV* human immunodeficiency virus, *HTA* health technology assessment, *IDU* injecting drug user, *LC* Liver Cirrhosis, *LYG* life-year gained, *MCBR* marginal cost-benefit ratio, *METH* methadone, *MHPP* Medical Heroin Prescription Program, *MMT* methadone maintenance treatment, *NA* not applicable, *NAL* naltrexone, *NHS* National Health Service, *NR* not reported, *NTX* extended release naltrexone, *NZ* New Zealand, *outpx* outpatient, *PSS* Personal & Social services, *QALY* quality-adjusted life-year, *RCT* randomised controlled trial, *SA* sensitivity analysis, *SMC* Scottish Medicines Consortium, *tx* treatment, *UK* United Kingdom, *US* United States of America

^a^Design as described by authors

### Interventions and populations considered

The interventions evaluated were established treatments in opioid dependence, including methadone maintenance treatment, buprenorphine maintenance treatment, medical heroin prescription, and buprenorphine combined with naloxone. Two US studies investigated the effect of expanding existing methadone maintenance treatment programmes. Studies compared between active treatments (for example, methadone maintenance treatment versus buprenorphine maintenance treatment), or between active treatment and no treatment or placebo (Table 2).

Three of the models did not report their baseline population. Of those studies that did report the baseline population, most used a hypothetical cohort of opioid-dependent patients (Table 2).

### Evaluation type

**Fig. 2:**
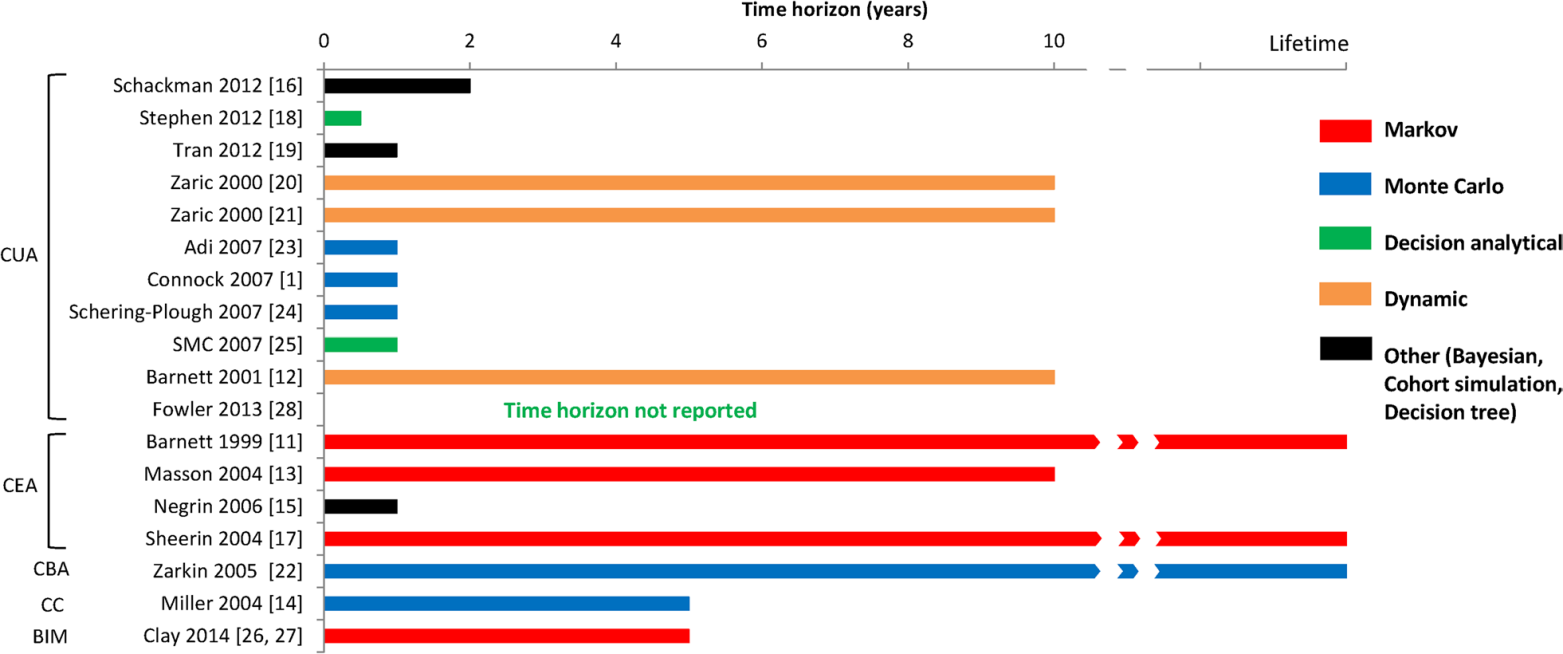
Modelling approaches, time horizons and evaluation types used in the included models. *BIM* budget impact, *CBA* cost-benefit analysis, *CC* cost comparison, *CEA* cost-effectiveness analysis, *CUA* cost-utility analysis

Figure 2 summarises the evaluation types, time horizons and modelling approaches in graphical form. The most common form of evaluation was a cost-utility analysis reporting the cost per quality-adjusted life-year (QALY) gained, followed by cost-effectiveness analysis reporting cost per life-year gained/saved. No other type of evaluation was used in more than one study (Table 2; Fig. 2).

Cost-utility evaluation was employed in 11/18 studies, and cost-effectiveness evaluation in 4/18 studies. The remaining three studies included a cost-benefit evaluation, a budget impact analysis, and a cost comparison (one study each).

### Assessing studies using an economic appraisal checklist

**Fig. 3:**
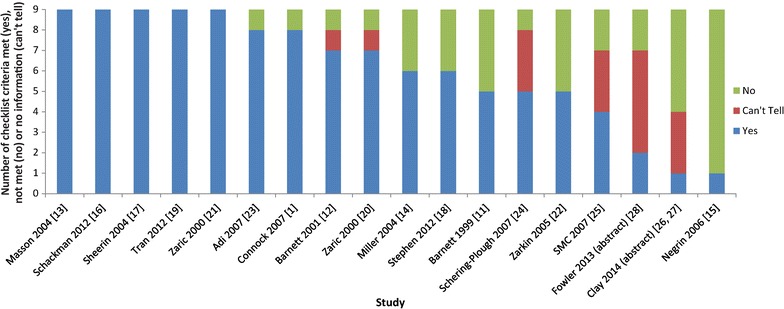
Assessing studies using an economic appraisal checklist

The results of the economic appraisal checklist are shown in Fig. 3 and Additional file 3. The two abstracts are included for completeness, but the limited space available in an abstract is likely to have restricted their ability to report full information on the models and the assessment should therefore be interpreted with caution. Of the 16 studies reported in journals or HTA reports, more than half (9/16) scored ‘Yes’ on at least seven of the nine questions on the checklist, and almost all (14/16) scored ‘Yes’ on at least five of the nine questions. Only five studies scored ‘Yes’ on all nine questions (Fig. 3).

### Country and perspective

The most commonly modelled countries were the US and the UK (Table 2). Eleven of the models were Canadian or US-based and one of the ten US studies did not explicitly report its perspective. Six US studies were from the perspective of the healthcare provider, and three from a societal perspective. The study based in Canada took a societal perspective by including costs associated with crime and considering the proportion of patients in employment [14].

Four studies used a UK National Health Service (NHS) perspective. Of these, three also included a societal perspective as a secondary analysis, although one of these reported no details of the societal perspective analysis. All four UK models were HTA reports.

Of the three remaining models, one was from the perspective of Spanish drug treatment centres, one from the perspective of the New Zealand healthcare system, and one from the perspective of the Vietnamese healthcare system.

Studies conducted from a societal perspective included costs borne outside the healthcare system, such as out-of-pocket costs incurred by patients, effects on employment and productivity, and the impact of criminal activity on the criminal justice system and on victims of crime.

### Time horizons

The time horizons varied widely, ranging from 6 months to a lifetime. Seven studies had short time horizons (6 months to a year), seven had time horizons of 2–10 years, and three models had a lifetime horizon. One abstract did not report the time horizon used (Table 2; Fig. 2).

### Modelling approaches

The 18 models used a range of different modelling approaches reported by authors. There did not seem to be any single approach that emerged as a preferred standard.

The most commonly used approaches were Markov modelling, which was used by four of the included models, and decision tree modelling with Monte Carlo simulations, which was used by three models.

Two further models used Monte Carlo simulations. Of the remaining nine, three were decision-analytical models, three were dynamic transmission models using epidemiological data on HIV prevalence, one used a decision tree, one used cohort simulation, and one used a Bayesian approach (Table 2; Fig. 2).

Decision-analysis trees are simple forms of health economic models that aim to represent clinical pathways over time and allows synthesis of evidence to estimate costs and effectiveness. These models are best suited to modelling acute conditions or short-term interventions. However, decision-analysis trees are generally too simple to be used for the modelling of situations where there are multiple alternative actions (for example, treatment pathways, drug options) that are encountered in complex or chronic conditions (such as long-term management of addiction) or in cases where events may be repeated (such as treatment cycles).

Markov models are particularly suited to modelling repeated events and/or progression of disease. In Markov models, there are a finite set of health states (for example, in treatment, illicit drug user, non-user not on treatment) and individuals move between these health states over a discrete time period, known as a Markov cycle and according to a set of transition probabilities (describing the probability of moving from one health state to another). By attaching costs and outcomes to the health states, and running the model over a number of cycles, the long term costs and outcomes for hypothetical cohorts can be estimated. In reality, the transitions/probabilities may vary based on individual patient treatment history, and Monte Carlo simulations may be used. These simulations use repeated random calculations to obtain a distribution of a particular outcome and take account of different treatment histories or varying transition probabilities.

### Outcomes measures and data sources

The data used in the models for outcomes and resource use were mainly derived from published literature, clinical trials, meta-analyses or indirect comparisons. The budget impact analysis used a health claims database (Table 2).

For those studies that reported details of the clinical outcomes used as model inputs, the choice of outcomes used depended on the scope of the economic evaluations. In all cases outcomes associated with maintenance treatment were either based on impacts on mortality, retention in the maintenance programme or successful detoxification and cessation of the maintenance treatment (Additional file 4). Other clinical outcomes/clinical effectiveness outcomes used were related to whether an individual was taking illicit drugs while on maintenance treatment, or while off treatment. Five economic evaluations considered the impact of needle-sharing and sexual behaviour of IDU on the spread of HIV, acquired immunodeficiency syndrome (AIDS) and HCV disease [12, 17, 19–21].

The impact of disease and treatment on quality of life is an important indicator of outcomes. When this is combined with clinical outcomes such as life-years gained, QALYs can be calculated. This outcome measure is recommended by HTA authorities [29–32]. The results of this review highlight the lack of evidence in this area of modelling. Additional file 5 summarises the sources of utility data used in the models reviewed. While utility weights are reported for HIV, AIDS and HCV, these are not specific to individuals with heroin abuse. Of the 11 studies which used a cost-utility approach and QALYs, four economic evaluations derived utility weights from a UK panel study which used general population members to make valuations of given health states using a standard gamble method (an approach recommended by NICE). One economic evaluation used mapping of a generic psychometric scale to QALY-based estimates [19]. For the remaining studies, assumptions were made based on plausible estimates in other diseases.

Costs included in the economic evaluations are summarised in Additional file 6. These included direct medical costs, which comprised all consumption of resources resulting from maintenance treatment (such as drug costs, healthcare resources); and direct non-medical costs such as staff costs, capital and building costs. The far-reaching consequences of heroin use, namely wider societal costs such as the costs of crime, employment, and support services have been included in only a limited number of studies. Only one study included costs to patients such as transport costs and their time spent travelling to and from treatment centres. The studies that included societal costs in the primary analysis varied widely in the type of costs they included; Schackman et al. [16] included only patient costs (travel, visit time and transport costs), Miller et al. [14] included the costs of criminal activity; Zarkin et al. [22] included criminal costs and productivity; and Stephen et al. [18] included criminal costs and productivity costs for both victim and crime perpetrator. A further two studies included societal costs as part of an additional analysis: for both, the costs included were criminal justice service costs [1, 23]. One further study mentioned an additional analysis including societal costs but reported no details [25].

**Fig. 4:**
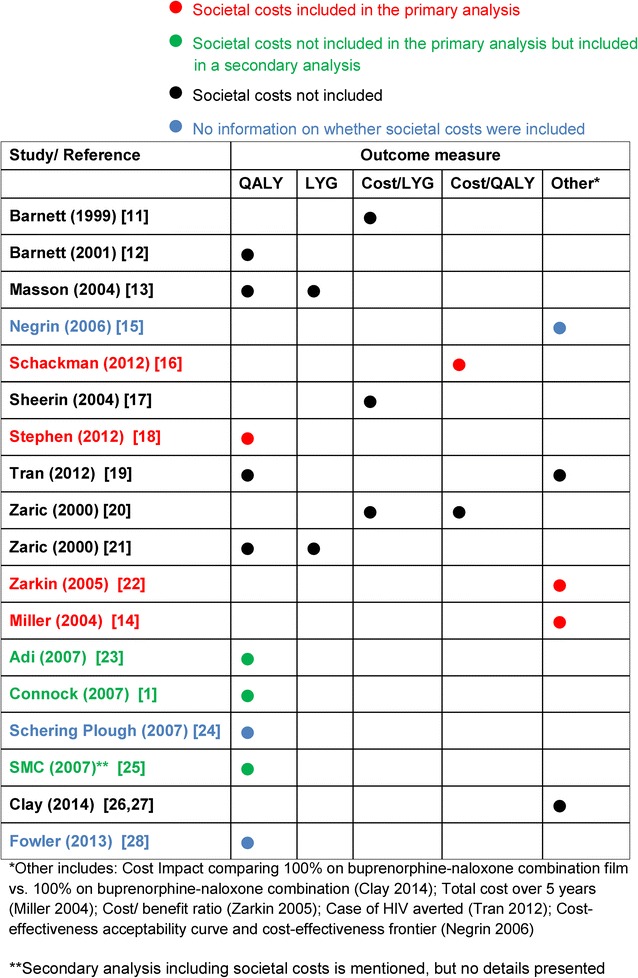
Overview of outcomes and costs considered. *HIV* human immunodeficiency virus, *LYG* life-years gained, *QALY* quality-adjusted life-years

Figure 4 summarises the outcome measures used in the studies and whether societal costs were included in the primary analysis. Only four studies included societal costs in the primary analysis [14, 16, 18, 22]. The most commonly used outcome measure was the QALY.

### Sensitivity analyses

A range of different types of sensitivity analyses have been reported in existing models in order to test the robustness of results. Deterministic and probabilistic analyses have been used.

In general, the results from the models did not vary significantly for the parameters tested in the various forms of sensitivity analyses, and ICERs usually remained within cost-effectiveness thresholds. However, the parameters which did impact on the results included treatment completion rates, health-related quality of life, and costs associated with criminal justice services.

## Discussion

Opioid dependence is typically a chronic condition with a dynamic and variable course. Individuals may have periods of illicit drug use interspersed with periods of treatment and periods of abstinence, and outcomes vary greatly from one individual to another. Opioid dependence also has complex effects on wider society, for example on the criminal justice system and victims of crime, so economic models should aim to capture these aspects in order to reflect the decision-making framework.

To our knowledge, this systematic review is the first to evaluate economic modelling studies conducted in opioid agonist therapy for non-prescription opioid dependence.

Several different modelling approaches have been reported, each with its own strengths and limitations. Decision-tree models typically accepted by HTA bodies are simple to construct and useful where short time horizons are appropriate and the estimation of outcomes is straightforward, but they do not easily capture time dependency or recurrent events in chronic or complex diseases such as heroin addiction. However, decision-tree models can be adapted to better reflect aspects of time dependency and/or longer term outcomes. An example of this is incorporation of Monte Carlo simulations, as was done in two of the HTA models reported [1, 23].

Other approaches that overcome the issues associated with modelling long-term or chronic conditions with discrete health states include cohort models or Markov models. In this type of model, patients are in one of a number of finite health states, which are mutually exclusive and represent clinically and economically important events. Movements between health states are determined by transition probabilities. Decision-tree models, cohort models and Markov models are favoured by HTA agencies for their simplicity of construction and analysis. However, the transition probability of moving from one disease state to another is independent of the patient’s disease history, and there is a fixed cycle time before a patient is eligible to move into a new state. These are potentially important limitations in opioid dependence, as transitions are affected by individual patient characteristics, previous behaviour and treatment history [22]. Ways to address these limitations within the Markov/cohort framework include using additional health states to account for disease history (as long as the number of states does not become too cumbersome), or the transition probabilities can be made time-dependent [1].

Patient-level simulation models such as dynamic models and Monte Carlo simulations can also be used to capture stochastic variation in outcomes between individuals, and can take account of heterogeneity in factors such as demographic characteristics and previous history. Their main deficiency is lack of transparency with regard to data inputs and how they are combined within the model, coupled with the prolonged time taken to run sensitivity analyses [1].

Dynamic models, used by three studies identified in this review [12, 20, 21] to evaluate population effects associated with needle sharing, allow internal feedback loops and time delays that permit the modelling of health changes across entire populations or systems. They are well established in the study of infectious disease transmission through populations [1].

Four studies identified in this review used Monte Carlo simulation [1, 22–24], and a further study used it for the sensitivity analyses [14]. However, these types of models are known to produce different results from those of static models, and the direction of the results may be unpredictable compared with models such as decision trees or Markov models. Comparisons of results between dynamic models and static models cannot easily be made. As a result, dynamic models may not be accepted by HTA assessment groups, as noted in a systematic review and economic evaluation of methadone and buprenorphine for the management of opioid dependence in 2007 [1].

A limitation of our review is that it did not include health economic models based solely on data from the duration of a randomised controlled trial, i.e. those that did not extrapolate beyond the trial duration. Whilst this does represent another possible modelling approach, the short duration of such trials means it is unlikely to be suitable for modelling a chronic condition such as non-prescription opioid dependence.

Model parameters such as time horizons were reflected in the modelling approach. Time horizons in decision analytical models ranged from 6 months to 1 year while cohort/simulation models ranged from 5 years to a lifetime. Modelling guidelines [30] recommend that the time horizon of a model should be long enough to capture all important differences in costs or outcomes between two or more treatments or between treatment and no treatment. Both models used in NICE HTA appraisals were 1 year in length [1, 23]. As opioid dependence may require long-term or life-long treatment [4], longer time horizons are likely to be more appropriate for models of opioid dependence, so that a realistic picture of lifetime costs and benefits of treatment can be adequately presented.

The model inputs reflected the perspective taken. Costs included in the economic evaluations were mainly those relating to healthcare service costs associated with opioid agonist therapy, including costs to primary care services and hospital services. Wider societal costs, such as costs of drug-related crime, disorder and antisocial behaviour, and loss of productivity in the workplace, were included in some form in only a few of the models. Impacts on family and social networks have not been included in any model. Costs to the individual patient, such as out-of-pocket costs, the costs related to premature death, drug-related illness and the loss of earnings through criminality/imprisonment, sickness, temporary or permanent unemployment and reduced educational attainment are also not routinely included in economic models. This may be because these types of costs are not ordinarily included in economic evaluations of healthcare interventions, so comparability with other interventions would be hampered if they were included. Indeed, HTA groups around the world have varying guidelines regarding costs to be included in a reference case for economic evaluations. In Sweden and The Netherlands, all relevant direct and indirect costs and revenues for treatment and ill health, irrespective of the payee, should be considered [29, 32]; in Norway, unrelated medical and non-medical costs should not be included [31]; in the UK a broader perspective on costs (beyond the NHS and Personal Social Services) may only be considered in exceptional circumstances [30]; while in Australia, PBAC mainly considers the costs of providing health care resources but may also consider costs and cost offsets of non-health care resources, although these might not be as influential in decision making as health care resources [33]. In the treatment of drug addiction, consistent omission of wider costs and benefits in the reported models means that the impact of effective maintenance treatments will not be fully captured; this will under-estimate the benefit of maintenance treatment to society. For example, reducing the spread of HIV will benefit non-drug users who will avoid HIV infection [11, 12] and is an important societal impact of maintenance treatment. In the US, total lifetime treatment cost for HIV based on new diagnoses in 2009 was estimated to be $16.6 billion [34].

The most common form of evaluation was cost-utility analysis, with an outcome measure of the number of QALYs gained. This may reflect the preference of HTA agencies for this type of analysis as it incorporates the value placed on health effects by society [30, 33]. However, there are limited data sources available for utility weights for substance abuse. Including the impact on quality of life is important because substance abuse is associated with significant co-morbidities and personal and social impacts, all of which should be considered when estimating the benefit of maintenance treatment. Thus, QALY weights should take account of reductions in health due to the disease of addiction itself and the impact of treatment on quality of life. In this review, only one source was available where the standard gamble method was used with members of the general public to obtain QALY weights for health states in substance abuse [16]. Choice-based preference measures to capture the value of health-related quality of life impacts are recommended by HTA groups such as NICE in the UK [30], PBAC in Australia [33] and HTA groups in European countries such as Norway [31], The Netherlands [29] and Sweden [32]. However, with addiction therapies and services the question could be asked about whose values should be used. Members of the public valuing vignettes developed by patients may be skewed by moral judgements, so perhaps people who have recovered from drug addiction might be the best-informed group.

## Conclusions

This systematic review identified 18 economic modelling studies in non-prescription opioid dependence reporting a range of modelling approaches. There appears to be no single standard emerging as the preferred approach and there are a number of advantages and disadvantages to the different modelling approaches, some of which can be overcome using advanced modelling techniques. These factors, together with acceptability to HTA agencies, need to be considered when selecting a model structure for economic evaluation of a new therapy indicated for the maintenance treatment of non-prescription opioid dependence.

The most common evaluation type was cost-utility analysis reporting the cost per QALY gained (11/18 models), reflecting the preference of HTA agencies for this form of evaluation, although the data on utility weights are limited. Typical outcomes include mortality and treatment retention. However, wider societal costs such as the cost of crime (expenditure by the criminal justice system in dealing with crimes committed, and cost consequences for the victims of crime), productivity, employment impacts, transmission of HIV and HCV and costs of treatment borne by patients (for example, transportation costs and value of time spent receiving treatment) are not typically included in health economic modelling. Given the wide ranging impact of illicit opioid abuse on the workplace, the healthcare system, and in the communities, including these non-typical costs may help present a more complete story of the economic burden.

This review identified some key elements that could form a standard framework and guide for future models, such as:

- Selecting a modelling approach that is able to capture the complexities of opioid use, allow for transitions to be affected by past behaviour and include transmission of HIV and other drug-related infectious diseases;
- Selecting a time horizon that is long enough to capture the impact of treatment and disease on patients in this chronic condition;
- Inclusion of societal consequences associated with heroin use that also have important economic consequences;
- Capturing the impact of disease and treatment-related health-related quality of life/utility specific to this patient population through appropriately sourced and valued health states.

The range of modelling approaches found in the literature could not easily be compared in terms of quality but the evidence does indicate that there is currently no single standard emerging as the preferred approach. Indeed, a number of the methodological approaches taken in different modelling studies could be used together to begin to build a framework for future models in this disease area. Furthermore, some of the disadvantages of current model structures could be overcome by using more advanced modelling techniques or choosing a different model approach. We propose that to develop a future model for the pharmacoeconomic evaluation of opioid agonist interventions, researchers should first identify the most robust model currently available, replicate the model, and then extend that model in terms of the range of inputs described above. Such a model should be applicable to a range of scenarios, e.g. intervention versus no intervention, comparison between interventions, changes over time, societal impact. Providing access to the model and the associated model code to the health care community could stimulate future dialogue and make it easier to recognise the strengths and weakness of different modelling approaches.

## Additional files

**Additional file 1.** Search strategy.

**Additional file 2.** Excluded publications.

**Additional file 3.** Assessing studies using an economic appraisal checklist.

**Additional file 4.** Summary of outcome parameters and source reported.

**Additional file 5.** Utility weights used.

**Additional file 6.** Summary of resource utilisation and costs.

## Abbreviations

AIDS: acquired immunodeficiency syndrome
CADTH: Canadian Agency for Drugs and Technologies in Health
DARE: Database of Abstracts of Reviews of Effects
DBS: deep brain stimulation
G-BA: Gemeinsamer Bundesausschuss
HCV: hepatitis C virus
HIV: human immunodeficiency virus
HTA: health technology assessment
IDU: injecting drug users
IQWIG: Institute for Quality and Efficiency in Heath Care
NHS: National Health Service
NHS EED: National Health Service Economic Evaluation Database
NICE: National Institute for Health and Clinical Excellence
PBAC: Pharmaceutical Benefits Advisory Committee
QALY: quality-adjusted life-year
SBU: Swedish Council on Health Technology Assessment
SMC: Scottish Medicines Consortium
TLV: Tandvards-och lakemedelsformansverket
US: United States of America
UK: United Kingdom
