# Supplementary material for: A systematic review of health economic models of opioid agonist therapies in maintenance treatment of non-prescription opioid dependence
Source: Addict Sci Clin Pract. 2017 Feb 24;12:6. doi: 10.1186/s13722-017-0071-3 (PMC5324212; doi:10.1186/s13722-017-0071-3)
Supplement: Supplementary file 1 — Additional file 1. Search strategy. [file 13722_2017_71_MOESM1_ESM.pdf]

Additional File 1: Search strategy

Cochrane Library

Cochrane Library includes Cochrane Database of Systematic Reviews (CDSR), Database of Abstracts of Reviews of Effects (DARE), Health Technology Assessments Database (HTA), NHS Economic Evaluations Database (NHSEED).

Searched 18/03/15 online at http://onlinelibrary.wiley.com/cochranelibrary/search/advanced. No date or language limits applied.

Search strategy:

#1 MeSH descriptor: [Methadone] this term only (930)

#2 MeSH descriptor: [Opiate Substitution Treatment] explode all trees (143)

#3 (methadon* or methadose or dolophine or dolafin):ti,ab (1665)

#4 MeSH descriptor: [Buprenorphine] this term only (678)

#5 (subutex or suboxone or buprenex or buprex or buprine or butrans):ti,ab (28)

#6 MeSH descriptor: [Morphine Derivatives] explode all trees (4766)

#7 (diamorphine or diacetylmorphine or acetomorphine or heroin):ti,ab (1057)

#8 (morphin* or morfin*):ti,ab (6724)

#9 (codein* or codipertussin or codyl or (methyl next/1 (morfine or morphine)) or methylmorfine or methylmorphine or pentus):ti,ab (861)

#10 (dihydrocodein* or codhydrin or codhydrine or codicontin or cohydrin or dehacodin or "dh codeine" or "di hydrin" or didrate or dihydrin or dihydroneopine or drocode or hydrocodeine or hydrocodin or nadein or nadeine or napacodin or novicodin or paracodein or paracodin or parzone or rapacodin or remedacen or "tiamon mono"):ti,ab (79)

#11 MeSH descriptor: [Naloxone] explode all trees (1577)

#12 (naloxon* or maloxone or nalone or nalonee or narcan or narcanti or narcon or narvcam or naxone or zynox):ti,ab (1534)

#13 (antaxone or celupan or nalerona or nalorex or naltrel or naltrexone or nemexin or nodict or nutrexon or phaltrexia or "re-via" or regental or revez or revia or trexan or vivitrex or vivitrol):ti,ab (1150)

#14 #1 or #2 or #3 or #4 or #5 or #6 or #7 or #8 or #9 or #10 or #11 or #12 or #13 (13022)

#15 MeSH descriptor: [Opioid-Related Disorders] explode all trees (1243)

#16 MeSH descriptor: [Substance-Related Disorders] this term only (2447)

#17 ((substance or opiate* or opioid* or heroin) near/2 (abus* or misus* or depend* or addict*)):ti,ab (3326)

#18 #15 or #16 or #17 (5364)

#19 #14 and #18 (1910)

Of total 1910 results 35 from NHSEED (37 from CDSR, 39 from DARE, 16 from HTA)

Medline and Medline In-Process

Searched 17/03/15 via OvidSP interface. No date or language limits applied.

Database: Ovid MEDLINE(R) In-Process & Other Non-Indexed Citations and Ovid MEDLINE(R) <1946 to Present>

Search strategy:

1 exp Methadone/ (10519)

2 Opiate Substitution Treatment/ (1139)

3 (methadon$ or methadose or dolophine or dolafin).ti,ab. (10640)

4 Buprenorphine/ (3804)

5 (subutex or suboxone or buprenex or buprex or buprine or butrans).ti,ab. (152)

6 exp Morphine Derivatives/ (44478)

7 (diamorphine or diacetylmorphine or acetomorphine or heroin).ti,ab. (11419)

8 (morphin$ or morfin$).ti,ab. (42696)

9 (codein$ or codipertussin or codyl or (methyl adj1 (morfine or morphine)) or methylmorfine or methylmorphine or pentus).ti,ab. (4095)

10 (dihydrocodein$ or codhydrin or codhydrine or codicontin or cohydrin or dehacodin or "dh codeine" or "di hydrin" or didrate or dihydrin or dihydroneopine or drocode or hydrocodeine or hydrocodin or nadein or nadeine or napacodin or novicodin or paracodein or paracodin or parzone or rapacodin or remedacen or "tiamon mono").ti,ab. (431)

11 exp Naloxone/ (22639)

12 (naloxon$ or maloxone or nalone or nalonee or narcan or narcanti or narcon or narvcam or naxone or zynox).ti,ab. (20693)

13 (antaxone or celupan or nalerona or nalorex or naltrel or naltrexone or nemexin or nodict or nutrexon or phaltrexia or "re-via" or regental or revez or revia or trexan or vivitrex or vivitrol).ti,ab. (5493)

14 or/1-13 (95395)

15 exp Opioid-Related Disorders/ (19384)

16 Substance-Related Disorders/ (79033)

17 ((substance or opiate$ or opioid$ or heroin) adj2 (abus$ or misus$ or depend$ or addict$)).ti,ab. (34420)

18 or/15-17 (108349)

19 "Costs and cost analysis"/ or cost-benefit analysis/ (101882)

20 exp "health care costs"/ (48119)

21 (cba or cea or cua or cma or cca).ti,ab. (34457)

22 "cost of illness"/ (18538)

23 (resource adj1 (utili?ation or "use" or usage)).ti,ab. (10231)

24 (Cost or costs or economic$ or pharmacoeconomi$ or price or prices or pricing or expense$ or expenditure or fiscal or funding or financial or finance or budget$).ti,ab. (595313)

25 (value adj2 money).ti,ab. (1051)

26 exp models, economic/ (10525)

27 ((statistical or economic$) adj3 model$).ti,ab. (17093)

28 monte carlo method/ (20655)

29 Markov chains/ (10196)

30 "decision trees"/ (8986)

31 ("monte carlo" or markov or "decision tree$").ti,ab. (45407)

32 quality-adjusted life years/ (7363)

33 (qaly or "Quality adjusted life" or Quality-adjusted-life).ti,ab. (7725)

34 or/19-33 (754444)

35 14 and 18 and 34 (1013)

36 35 not (animals/ not (human/ and animals/)) (975)

37 36 not (case reports or editorial or letter).pt. (941)

941 results.

Embase

Searched 18/03/15 via OvidSP interface. No date or language limits applied.

Database: Embase <1974 to 2015 Week 11>

Search strategy:

1 methadone/ (25599)

2 methadone treatment/ (3318)

3 (methadon$ or methadose or dolophine or dolafin).ti,ab. (13879)

4 buprenorphine/ (11736)

5 (subutex or suboxone or buprenex or buprex or buprine or butrans).ti,ab. (298)

6 diamorphine/ (19480)

7 (diamorphine or diacetylmorphine or acetomorphine or heroin).ti,ab. (15017)

8 morphine/ (86407)

9 morphine sulfate/ (6489)

10 (morphin$ or morfin$).ti,ab. (54053)

11 codeine/ (17702)

12 (codein$ or codipertussin or codyl or (methyl adj1 (morfine or morphine)) or methylmorfine or methylmorphine or pentus).ti,ab. (5478)

13 dihydrocodeine/ (2113)

14 (dihydrocodein$ or codhydrin or codhydrine or codicontin or cohydrin or dehacodin or "dh codeine" or "di hydrin" or didrate or dihydrin or dihydroneopine or drocode or hydrocodeine or hydrocodin or nadein or nadeine or napacodin or novicodin or paracodein or paracodin or parzone or rapacodin or remedacen or "tiamon mono").ti,ab. (588)

15 naloxone/ (36891)

16 (naloxon$ or maloxone or nalone or nalonee or narcan or narcanti or narcon or narvcam or naxone or zynox).ti,ab. (23997)

17 naltrexone/ (11554)

18 (antaxone or celupan or nalerona or nalorex or naltrel or naltrexone or nemexin or nodict or nutrexon or phaltrexia or "re-via" or regental or revez or revia or trexan or vivitrex or vivitrol).ti,ab. (6739)

19 or/1-18 (174123)

20 opiate addiction/ (11092)

21 substance abuse/ (41509)

22 heroin dependence/ (7844)

23 withdrawal syndrome/ (24766)

24 ((substance or opiate$ or opioid$ or heroin) adj2 (abus$ or misus$ or depend$ or addict$)).ti,ab. (44693)

25 or/20-24 (97207)

26 exp economic evaluation/ (222386)

27 health economics/ (34181)

28 exp "health care cost"/ (214021)

29 exp pharmacoeconomics/ (171842)

30 "cost benefit analysis"/ (66797)

31 "cost effectiveness analysis"/ (103734)

32 "cost utility analysis"/ (5877)

33 "cost minimization analysis"/ (2604)

34 (cba or cea or cua or cma or cca).ti,ab. (45101)

35 "cost of illness"/ (14847)

36 (resource adj1 (utili?ation or "use" or usage)).ti,ab. (15671)

37 (Cost or costs or economic$ or pharmacoeconomi$ or price or prices or pricing or expense$ or expenditure or fiscal or funding or financial or finance or budget$).ti,ab. (761127)

38 (value adj2 money).ti,ab. (1492)

39 statistical model/ (109711)

40 ((statistical or economic$) adj3 model$).ti,ab. (20607)

41 monte carlo method/ (23445)

42 Markov chains/ (59362)

43 decision support system/ (14254)

44 "decision tree"/ (6407)

45 probability/ (59362)

46 ("monte carlo" or markov or "decision tree$").ti,ab. (47868)

47 quality adjusted life year/ (13419)

48 (qaly or "Quality adjusted life" or Quality-adjusted-life).ti,ab. (11933)

49 or/26-48 (1282788)

50 19 and 25 and 49 (2004)

51 50 not (animals/ not (human/ and animals/)) (2002)

52 51 not (editorial or letter).pt. (1883)

53 limit 52 to embase (1598)

1598 results.

HTA websites

Search terms used in the search fields of the respective websites were those listed in the Inclusion/exclusion criteria.
