# Supplementary material for: A systematic review of health economic models of opioid agonist therapies in maintenance treatment of non-prescription opioid dependence
Source: Addict Sci Clin Pract. 2017 Feb 24;12:6. doi: 10.1186/s13722-017-0071-3 (PMC5324212; doi:10.1186/s13722-017-0071-3)
Supplement: Supplementary file 2 — Additional file 2. Excluded publications. [file 13722_2017_71_MOESM2_ESM.pdf]

Additional file 2. Excluded publications and reason for exclusion

Full reference	Reason for exclusion
Barnett PGH, S. S. The cost-effectiveness of methadone maintenance. Mt Sinai J Med 2000;67(5-6):365-74.	Review
Barnett PG. The cost-effectiveness of substance abuse treatment. Curr Psychiatry Rep 1999;1(2):166-71.	Review
Doran CM. Economic evaluation of interventions to treat opiate dependence: A review of the evidence. PharmacoEconomics 2008;26(5):371-93.	Review
Gastfriend DR. A pharmaceutical industry perspective on the economics of treatments for alcohol and opioid use disorders. Ann N Y Acad Sci 2014;1327:112-30.	Review
Krupitsky E. Injectable extended-release naltrexone for the prevention of relapse to opioid dependence following opioid detoxification. Neuropsychiatry 2012;2(4):355-62.	Review
Lintzeris N. Prescription of heroin for the management of heroin dependence: current status. CNS Drugs 2009;23(6):463-76.	Review
McCarty D. The cost effectiveness of treatment with extended-release naltrexone: A structured review across four studies. Alcohol Clin Exp Res 2012;36:342A	Review
Moore TJR, A.; Caulkins, J. P. The costs and consequences of three policy options for reducing heroin dependency. Drug Alcohol Rev 2007;26(4):369-78.	Review
Simoens SL, A.; Matheson, C.; Bond, C. Pharmaco-economics of community maintenance for opiate dependence: a review of evidence and methodology. Drug Alcohol Depend 2006;84(1):28-39.	Review
Vanagas GP, Z.; Bagdonas, E. Cost-utility analysis of methadone maintenance treatment: a methodological approach. Subst Use Misuse 2006;41(1):87-101.	Review
Goldschmidt P.G. A cost-effectiveness model for evaluating health care programs: application to drug abuse treatment. Inquiry 1976 13 (1): 29-47	Outdated, more than 30 years old
Sirotnik, K. A.; Bailey, R. C. A cost--benefit analysis for a multimodality heroin treatment project. International Journal of the Addictions. 1975 10(3): 443-451	Outdated, more than 30 years old
Byford SB, B.; Metrebian, N.; Groshkova, T.; Cary, M.; Charles, V.; Lintzeris, N.; Strang, J. Cost-effectiveness of injectable opioid treatment v. oral methadone for chronic heroin addiction. Br J Psychiatry 2013;203(5):341-9.	Economic evaluations alongside clinical study
Dijkgraaf MGvdZ, B. P.; de Borgie, C. A.; Blanken, P.; van Ree, J. M.; van den Brink, W. Cost utility analysis of co-prescribed heroin compared with methadone maintenance treatment in heroin addicts in two randomised trials. BMJ 2005;330(7503):1297.	Economic evaluations alongside clinical study
Doran CM. Buprenorphine, buprenorphine/naloxone and methadone maintenance: a cost-effectiveness analysis. Expert rev 2005;5(5):583-91.	Economic evaluations alongside clinical study
Doran CMS, M.; Digiusto, E.; O'Brien, S.; Mattick, R. P. Cost-effectiveness analysis of maintenance agonist treatments in the NEPOD. Expert rev 2006;6(4):437-46.	Economic evaluations alongside clinical study
Doran CMS, M.; Mattick, R. P.; Ali, R.; White, J.; Bell, J. Buprenorphine versus methadone maintenance: a cost-effectiveness analysis. Drug Alcohol Depend 2003;71(3):295-302.	Economic evaluations alongside clinical study
Harris AHG, E.; Ritter, A. J. A randomised trial of the cost effectiveness of buprenorphine as an alternative to methadone maintenance treatment for heroin dependence in a primary care setting. Pharmacoeconomics 2005;23(1):77-91.	Economic evaluations alongside clinical study
Harwood H, Hubbard RL, Collins JJ, et al. Cost Benefit of Drug Abuse Treatment. Research in Law and Policy Studies 1995;3:191-214.	Economic evaluations alongside clinical study

Maas JB, G.; Maskrey, V.; Pinto, H.; Holland, R. Economic evaluation: a comparison of methadone versus buprenorphine for opiate substitution treatment. Drug Alcohol Depend 2013;133(2):494-501.	Economic evaluations alongside clinical study
Marino VL, C. Buprenorphine/naloxone versus buprenorphine and methadone in heroin addiction detoxification: An Italian cost-utility analysis. Value Health 2011;14 (7):A293.	Economic evaluations alongside clinical study
National Drug and Alcohol Research Centre. National Evaluation of Pharmacotherapies for Opioid Dependence (NEPOD): Report of Results and Recommendations. Sidney, Australia: University of New South Wales, 2001.	Economic evaluations alongside clinical study
Nosyk BG, D. P.; Bansback, N. J.; Oviedo-Joekes, E.; Brissette, S.; Marsh, D. C.; Meikleham, E.; Schechter, M. T.; Anis, A. H. Cost-effectiveness of diacetylmorphine versus methadone for chronic opioid dependence refractory to treatment. CMAJ Canadian Medical Association Journal 2012;184(6):E317-28.	Economic evaluations alongside clinical study
Ruger JPC, M.; Mazlan, M.; Ng, N.; Schottenfeld, R. Cost-effectiveness of buprenorphine and naltrexone treatments for heroin dependence in Malaysia. PLoS One 2012;7(12):e50673.	Economic evaluations alongside clinical study
Russell CM, N. A cost-effectiveness analysis of extending methadone and buprenorphine-naloxone maintenance treatment of opioid dependence from eight to sixteen months. Value Health 2013;16 (3):A61.	Economic evaluations alongside clinical study
Sheerin IG, T.; Sellman, D.; Adamson, S.; Deering, D. Reduction in crime by drug users on a methadone maintenance therapy programme in New Zealand. N Z Med J 2004;117(1190):U795.	Economic evaluations alongside clinical study
Strang JM, J.; Cummins, M.; Farrell, M.; Finch, E.; Gossop, M.; Stewart, D.; Welch, S. Randomized trial of supervised injectable versus oral methadone maintenance: report of feasibility and 6-month outcome. Addiction 2000;95(11):1631-45.	Economic evaluations alongside clinical study
Taylor ML, L.; McKeganey, N. An economic analysis of the impact of crime and hospitalisation associated with different interventions for opioid abuse in the United Kingdom. Value Health 2011;14 (7):A291.	Economic evaluations alongside clinical study
Vanagas GP, Z.; Bagdonas, E. Cost-utility analysis of methadone maintenance treatment in Lithuania. Medicina (Kaunas) 2010;46(4):286-92.	Economic evaluations alongside clinical study
Warren EV, R.; Shearer, J.; Shanahan, M.; Wodak, A.; Dolan, K. Value for money in drug treatment: economic evaluation of prison methadone. Drug Alcohol Depend 2006;84(2):160-6.	Economic evaluations alongside clinical study
Barnett PGS, J. L.; Wong, W.; Haug, N. A.; Hall, S. M. Effect of incentives for medication adherence on health care use and costs in methadone patients with HIV. Drug Alcohol Depend 2009;100(1-2):115-21.	Non-CEA model based cost analysis
Baser OC, M.; Fiellin, D. A.; Gastfriend, D. R. Cost and utilization outcomes of opioid-dependence treatments. Am J Manag Care 2011;17 Suppl 8:S235-48.	Non-CEA model based cost analysis
Geitona MC, V.; Petratos, P. Economic evaluation of opioid substitution treatment in Greece. Heroin Addiction and Related Clinical Problems 2012;14(3):77-88.	Non-CEA model based cost analysis
Geitona MC, V.; Petratos, P.; Androutsou, L. Economic evaluation of opioid substitution treatment (OST) in Greece. Value Health 2012;15 (7):A512.	Non-CEA model based cost analysis
Martinez-Raga JC, M. A.; Gonzalez Saiz, F.; Onate, J. Budgetary impact analysis of buprenorphine/naloxone (suboxone) in opioid maintenance treatment in Spain. Value Health 2011;14 (7):A289.	Non-CEA model based cost analysis
Martinez-Raga JG-S, F.; Onate, J.; Oyaguez, I.; Sabater, E.; Casado, M. A. Budgetary impact analysis of buprenorphine-naloxone combination (Suboxone) in Spain. Health Econ Rev 2012;2(1):3.	Non-CEA model based cost analysis
Martinez-Raga JGS, F.; Pascual, C.; Casado, M. A.; Sabater Torres, F. J. Suboxone (buprenorphine/naloxone) as an agonist opioid treatment in Spain: a budgetary impact analysis. Eur Addict Res 2010;16(1):31-42.	Non-CEA model based cost analysis
Reutsch CS, C. A.; Tkacz, J. Cost-effectiveness of medication-replacement therapy with buprenorphine among opioid-dependent patients: Results from a retrospective analysis of health plan membership claims data. J Manage Care Pharm 2009;15 (7):578-79.	Non-CEA model based cost analysis

Rosenheck R, Kosten T. Buprenorphine for opiate addiction: potential economic impact. Drug Alcohol Depend 2001;63(3):253-62.	Non-CEA model based cost analysis
Ruetsch CT, J. Buprenorphine-medication assisted treatment: A retrospective analysis of health plan data. Am J Addict 2010;19 (4):368-69.	Non-CEA model based cost analysis
Tkacz JU, H.; Leader Jr, D.; Ruetsch, C. The effectiveness of buprenorphine-medication assisted treatment among aetna's opioid dependent members: An analysis of health care costs and service utilization. Value Health 2011;14 (3):A191.	Non-CEA model based cost analysis
Zaric GSB, A. W.; Varenbut, M.; Daiter, J. M. The cost of providing methadone maintenance treatment in Ontario, Canada. Am J Drug Alcohol Abuse 2012;38(6):559-66.	Non-CEA model based cost analysis
