# Supplementary material for: A systematic review of health economic models of opioid agonist therapies in maintenance treatment of non-prescription opioid dependence
Source: Addict Sci Clin Pract. 2017 Feb 24;12:6. doi: 10.1186/s13722-017-0071-3 (PMC5324212; doi:10.1186/s13722-017-0071-3)
Supplement: Supplementary file 3 — Additional file 3. Assessing studies using an economic appraisal checklist. [file 13722_2017_71_MOESM3_ESM.pdf]

Additional File 3. Assessing Studies using an Economic Appraisal Checklist

Study	Question [a]								
	Q 1	Q 2	Q 3	Q 4	Q 5	Q 6	Q 7	Q 8	Q 9
Adi (2007) [1]	Yes	Yes	Yes[b]	Yes	Yes	Yes	No	Yes	Yes
Barnett (1999) [2]	Yes	Yes	Yes	No	No	No	Yes	No	Yes
Barnett (2001) [3]	Yes	Yes	No	Yes	Yes	Can't tell	Yes	Yes	Yes
Clay (2014) (abstract) [4, 5]	Yes	No	No	Can't tell	Can't tell	Can't tell	No	No	No
Connock (2007) [6]	Yes	Yes	Yes	Yes	Yes	Yes	No	Yes	Yes
Fowler (2013) (abstract) [7]	Yes	No	No	Can't tell	Can't tell	Can't tell	Can't tell	Can't tell	Yes
Masson (2004) [8]	Yes	Yes	Yes	Yes	Yes	Yes	Yes	Yes	Yes

Study	Question [a]								
	Q 1	Q 2	Q 3	Q 4	Q 5	Q 6	Q 7	Q 8	Q 9
Miller (2004) [9]	Yes	Yes	Yes	Yes	Yes	No	No	No	Yes
Negrin (2006) [10]	No	No	Yes	No	No	No	No	No	No
Schackman (2012) [11]	Yes	Yes	Yes	Yes	Yes	Yes	Yes	Yes	Yes
Schering-Plough (2007) [12]	Yes	Yes	Yes[c]	Can't tell	Can't tell	Can't tell	No	Yes	Yes
Sheerin (2004) [13]	Yes	Yes	Yes	Yes	Yes	Yes	Yes	Yes	Yes
SMC (2007) [14]	Yes	No	Yes	No	Can't tell	Can't tell	Can't tell	Yes	Yes
Stephen (2012) [15]	Yes	Yes	Yes	Yes	Yes	No	No	Yes	No
Tran (2012) [16]	Yes	Yes	Yes	Yes	Yes	Yes	Yes	Yes	Yes
Zaric (2000) [17]	Yes	No	Yes	Yes	Yes	Can't tell	Yes	Yes	Yes

Study	Question [a]								
	**Q 1**	**Q 2**	**Q 3**	**Q 4**	**Q 5**	**Q 6**	**Q 7**	**Q 8**	**Q 9**
Zaric (2000) [18]	Yes	Yes	Yes	Yes	Yes	Yes	Yes	Yes	Yes
Zarkin (2005) [19]	Yes	No	Yes	Yes	No	No	Yes	No	Yes

[a] Questions:

1. Was a well-defined question posed in an answerable form?

2. Was a comprehensive description of the competing alternatives given?

3. Was there evidence that the programme's effectiveness had been established?

4. Were all the important and relevant outcomes and costs for each alternative identified?

5. Were outcomes and costs measured accurately, in appropriate units prior to evaluation?

6. Were the outcomes and costs valued credibly?

7. Were the outcomes and costs adjusted for different times at which they occurred (discounting)?

8. Was an incremental analysis of the outcomes and costs of alternatives performed?

9. Was a sensitivity analysis performed?

[b] quality of included randomised controlled trials was 'poor to moderate'

[c] from one randomised controlled trial

References

1. Adi Y, Juarez-Garcia A, Wang D, Jowett S, Frew E, Day E et al. Oral naltrexone as a treatment for relapse prevention in formerly opioid-dependent drug users: a systematic review and economic evaluation. Health Technol Assess. 2007;11:iii-iv, 1-85.
2. Barnett PG. The cost-effectiveness of methadone maintenance as a health care intervention. Addiction. 1999;94:479-88.
3. Barnett PG, Zaric GS, Brandeau ML. The cost-effectiveness of buprenorphine maintenance therapy for opiate addiction in the United States. Addiction. 2001;96:1267-78.
4. Clay E, Kharitonova E, Ruby J, Aballea S, Zah V. Medicaid population budget impact analysis of buprenorphine/naloxone film and tablet formulation. Value Health. 2014;17:A212.
5. Clay E, Khemiri A, Ruby J, Aballea S, Zah V. A studies-based private insurance budget impact analysis of buprenorphine/naloxone film and tablet formulations. Value Health. 2014;17:A213.

6. Connock M, Juarez-Garcia A, Jowett S, Frew E, Liu Z, Taylor RJ et al. Methadone and buprenorphine for the management of opioid dependence: a systematic review and economic evaluation. Health Technol Assess. 2007;11:1-171, iii-iv.

7. Fowler J, Emerson J, Allen A, Dilley S, Gideonse N, Rieckmann T et al. Buprenorphine vs methadone for maintenance of opioid addiction during pregnancy: A cost-effectiveness analysis. Am J Obstet Gynecol. 2013;208 (1 SUPPL.1):S65-6.

8. Masson CL, Barnett PG, Sees KL, Delucchi KL, Rosen A, Wong W et al. Cost and cost-effectiveness of standard methadone maintenance treatment compared to enriched 180-day methadone detoxification. Addiction. 2004;99:718-26.

9. Miller CL, Schechter MT, Wood E, Spittal PM, Li K, Laliberte N et al. The potential health and economic impact of implementing a medically prescribed heroin program among Canadian injection drug users. Int J Drug Policy. 2004;15:259-63.

10. Negrin MA, Vazquez-Polo FJ. Bayesian cost-effectiveness analysis with two measures of effectiveness: the cost-effectiveness acceptability plane. Health Econ. 2006;15:363-72.

11. Schackman BR, Leff JA, Polsky D, Moore BA, Fiellin DA. Cost-effectiveness of long-term outpatient buprenorphine-naloxone treatment for opioid dependence in primary care. J Gen Intern Med. 2012;27:669-76.

12. Schering-Plough. Manufacturer's submission. Cited in Connock et al. Health Technol Assess. 2007;11:1-171, iii-iv.

13. Sheerin IG, Green FT, Sellman JD. What is the cost-effectiveness of hepatitis C treatment for injecting drug users on methadone maintenance in New Zealand? Drug Alcohol Rev. 2004;23:261-72.

14. Scottish Medicines Consortium. Buprenorphine/naloxone 2mg/0.5mg, 8/2mg sublingual tablet (Suboxone). No. 355/07. 2007. https://www.scottishmedicines.org.uk/files/buprenorphine_naloxone_sublingual_tablet__Suboxone__355-07_.pdf. Accessed 3 Jun 2015.

15. Stephen JH, Halpern CH, Barrios CJ, Balmuri U, Pisapia JM, Wolf JA et al. Deep brain stimulation compared with methadone maintenance for the treatment of heroin dependence: a threshold and cost-effectiveness analysis. Addiction. 2012;107:624-34.

16. Tran BX, Ohinmaa A, Duong AT, Nguyen LT, Vu PX, Mills S et al. The cost-effectiveness and budget impact of Vietnam's methadone maintenance treatment programme in HIV prevention and treatment among injection drug users. Glob Public Health. 2012;7:1080-94.

17. Zaric GS, Barnett PG, Brandeau ML. HIV transmission and the cost-effectiveness of methadone maintenance. Am J Public Health. 2000;90:1100-11.

18. Zaric GS, Brandeau ML, Barnett PG. Methadone maintenance and HIV prevention: A cost-effectiveness analysis. Manage Sci. 2000;46:1013-31.

19. Zarkin GA, Dunlap LJ, Hicks KA, Mamo D. Benefits and costs of methadone treatment: results from a lifetime simulation model. Health Econ. 2005;14:1133-50.
