# Supplementary material for: A systematic review of health economic models of opioid agonist therapies in maintenance treatment of non-prescription opioid dependence
Source: Addict Sci Clin Pract. 2017 Feb 24;12:6. doi: 10.1186/s13722-017-0071-3 (PMC5324212; doi:10.1186/s13722-017-0071-3)
Supplement: Supplementary file 4 — Additional file 4. Summary of outcome parameters and source reported. [file 13722_2017_71_MOESM4_ESM.pdf]

Additional File 4. Summary of outcome parameters and source reported

Reference	Outcome	Source
Barnett (1999) [1]	Annual mortality rate in 25 year olds	US life tables
	Mortality rates on MMT	Based on a Swedish study comparing two groups of heroin addicts with different access to methadone
	Mortality rates not on MMT	Based on a Swedish study comparing two groups of heroin addicts with different access to methadone
Barnett (2001) [2]	For untreated population, MMT, and BMT:	
	* Annual number of injections	Combination references sourced via literature review, details of which are reported in another paper by same authors. Assumptions also used, e.g. % in MMT in a given year that will detoxify successfully and
	* % of injections that are shared	
	* Annual number of sexual partners	

Reference	Outcome	Source
	* Mortality rate from non-HIV causes	cease further IDU
	* Success detox and cease treatment	
	Short term efficacy of MMT and BMT	Meta-analysis on short term use, assumption that short term efficacy the same as long term outcomes
Masson (2004) [3]	Mean days of heroin use (self-reported)	Based on RCT comparing MMT to 180-day psychosocially enriched detoxification for treatment of opioid dependence
	Urine toxicology screening	
Negrin (2006) [4]	Treatment retention rates	12 month data obtained from a multicentre study comparing three MMT programmes in five drug treatment centres in Barcelona
Schackman (2012) [5]	Treatment retention rates on BMT	Derived from prospective observational cohort study of 53 long term opioid users treated with office-based BUP/NAL in primary care setting.
	Illicit drug use while on BMT	

Reference	Outcome	Source
	Using illicit drugs while off treatment	Based on evidence from the National Treatment Outcome Research Study (NTORS). This was a study carried out by the National Addiction Centre in England and Wales between the years 1995 and 2000 - the largest UK drug treatment outcome study at the time. This study followed up the clients of a variety of drug treatment service types for up to five years, examining a wide range of outcome measures.
Sheerin (2004) [6]	MMT treatment outcomes	Based on published reference which reports on addict death rates during a four year posttreatment follow-up.
	HCV disease	Full details not reported in this paper. However, sources for HCV transition probabilities are reported in another reference by same authors on the costs of not treating hepatitis C virus infection in injecting drug users in New Zealand
Stephen	Completing MMT	Meta-analysis based on 15 studies MMT

Reference	Outcome	Source
(2012) [7]		
	Completing with clean urine	
	Complications on DBS	Published reference on a decision analysis study of deep brain stimulation compared with bariatric surgery for the treatment of morbid obesity.
Tran (2012) [8]	MMT treatment outcomes	Longitudinal cohort study across 6 MMT clinics in Vietnam
	Drug-use behaviour	
	New cases of HIV averted due to MMT (estimates based on the number of risk acts [unsafe sex, drug injection, etc], percentage of protected acts and current prevalence of HIV)	Based on a published model (UNAIDS Mode of Transmission model) in which short term estimates of HIV incidence by transmission are modelled.
	Outcomes related to ART (including	Combination of publically available reference sources and healthcare

Reference	Outcome	Source
	retention on treatment, drop-off rates, viral suppression rates)	databases.
Zaric (2000) [9]	MMT outcomes	Based on sources found in literature based and reported in another reference by same authors. Assumptions were also made where multiple sources were found
	Mortality rates on MMT	
	HIV transmission rates	
Zaric (2000) [10]	MMT treatment outcomes	Based on published literature
	Sexual behaviour in IDU and non IDU	
	Injection drug behaviour	
	HIV transmission rates	
	Disease progression to AIDS	
Zarkin (2005)	Heroin use	Estimated based on published literature

Reference	Outcome	Source
[11]		
	Heroin treatment	Estimated based on published literature
	Crime rates	Estimated based on published literature
	Employment rates	Estimated based on published literature
Miller (2004) [12]	Outcomes on MHPP	National statistics, open cohort study of IDU and Swiss Heroin study
Adi (2007) [13]	Treatment retention rates on NAL	Meta-analysis of 5 RCTs
	Level and nature of drug misuse	One RCT
	Number injecting versus non-injecting	Based on evidence from the National Treatment Outcome Research Study (NTORS). This was a study carried out by the National Addiction Centre in England and Wales between the years 1995 and 2000 - the largest UK drug treatment outcome study at the time. This study followed up the clients of a variety of drug treatment service

Reference	Outcome	Source
		types for up to five years, examining a wide range of outcome measures.
Connock (2007) [14]	Treatment retention rates on BMT and MMT	Cochrane systematic review of buprenorphine maintenance versus placebo or methadone maintenance for opioid dependence
	Level and nature of drug misuse	
	Number injecting versus non-injecting	Based on evidence from the National Treatment Outcome Research Study (NTORS). This was a study carried out by the National Addiction Centre in England and Wales between the years 1995 and 2000 - the largest UK drug treatment outcome study at the time. This study followed up the clients of a variety of drug treatment service types for up to five years, examining a wide range of outcome measures.
Schering-Plough (2007)	Treatment retention rates on BMT	Based on a randomized double-blind trial with 405 opioid-dependent patients in which buprenorphine was compared to methadone

Reference	Outcome	Source
[15]		maintenance therapy

BMT, buprenorphine maintenance treatment; BUP/NAL, buprenorphine-naloxone combination; DBS, deep brain stimulation; HCV, hepatitis C virus; HIV, human immunodeficiency virus; IDU, injecting drug user; MHPP, Medical Heroin Prescription Program; MMT, methadone maintenance treatment; NAL, naltrexone; RCT, randomised controlled trial; UK, United Kingdom; US, United States

References

1. Barnett PG. The cost-effectiveness of methadone maintenance as a health care intervention. Addiction. 1999;94:479-88.
2. Barnett PG, Zaric GS, Brandeau ML. The cost-effectiveness of buprenorphine maintenance therapy for opiate addiction in the United States. Addiction. 2001;96:1267-78.
3. Masson CL, Barnett PG, Sees KL, Delucchi KL, Rosen A, Wong W et al. Cost and cost-effectiveness of standard methadone maintenance treatment compared to enriched 180-day methadone detoxification. Addiction. 2004;99:718-26.
4. Negrin MA, Vazquez-Polo FJ. Bayesian cost-effectiveness analysis with two measures of effectiveness: the cost-effectiveness acceptability plane. Health Econ. 2006;15:363-72.
5. Schackman BR, Leff JA, Polsky D, Moore BA, Fiellin DA. Cost-effectiveness of long-term outpatient buprenorphine-naloxone treatment for opioid dependence in primary care. J Gen Intern Med. 2012;27:669-76.

6. Sheerin IG, Green FT, Sellman JD. What is the cost-effectiveness of hepatitis C treatment for injecting drug users on methadone maintenance in New Zealand? Drug Alcohol Rev. 2004;23:261-72.

7. Stephen JH, Halpern CH, Barrios CJ, Balmuri U, Pisapia JM, Wolf JA et al. Deep brain stimulation compared with methadone maintenance for the treatment of heroin dependence: a threshold and cost-effectiveness analysis. Addiction. 2012;107:624-34.

8. Tran BX, Ohinmaa A, Duong AT, Nguyen LT, Vu PX, Mills S et al. The cost-effectiveness and budget impact of Vietnam's methadone maintenance treatment programme in HIV prevention and treatment among injection drug users. Glob Public Health. 2012;7:1080-94.

9. Zaric GS, Barnett PG, Brandeau ML. HIV transmission and the cost-effectiveness of methadone maintenance. Am J Public Health. 2000;90:1100-11.

10. Zaric GS, Brandeau ML, Barnett PG. Methadone maintenance and HIV prevention: A cost-effectiveness analysis. Manage Sci. 2000;46:1013-31.

11. Zarkin GA, Dunlap LJ, Hicks KA, Mamo D. Benefits and costs of methadone treatment: results from a lifetime simulation model. Health Econ. 2005;14:1133-50.

12. Miller CL, Schechter MT, Wood E, Spittal PM, Li K, Laliberte N et al. The potential health and economic impact of implementing a medically prescribed heroin program among Canadian injection drug users. Int J Drug Policy. 2004;15:259-63.

13. Adi Y, Juarez-Garcia A, Wang D, Jowett S, Frew E, Day E et al. Oral naltrexone as a treatment for relapse prevention in formerly opioid-dependent drug users: a systematic review and economic evaluation. Health Technol Assess. 2007;11:iii-iv, 1-85.

14. Connock M, Juarez-Garcia A, Jowett S, Frew E, Liu Z, Taylor RJ et al. Methadone and buprenorphine for the management of opioid dependence: a systematic review and economic evaluation. Health Technol Assess. 2007;11:1-171, iii-iv.

15. Schering-Plough. Manufacturer's submission. Cited in Connock et al. Health Technol Assess. 2007;11:1-171, iii-iv.
