# Supplementary material for: A systematic review of health economic models of opioid agonist therapies in maintenance treatment of non-prescription opioid dependence
Source: Addict Sci Clin Pract. 2017 Feb 24;12:6. doi: 10.1186/s13722-017-0071-3 (PMC5324212; doi:10.1186/s13722-017-0071-3)
Supplement: Supplementary file 5 — Additional file 5. Utility weights used. [file 13722_2017_71_MOESM5_ESM.pdf]

Additional File 5. Utility weights used

Reference	Details	Source/Notes
Barnett (2001) [1]	Utility adjustments made for asymptomatic HIV, AIDs, maintenance treatment and quality of life of IDU.	Utility for HIV and AIDs sourced from published literature. No utilities available for substance disorders so these values are assumed. The authors justify these utility adjustments by comparing to other conditions which limit activities.
Masson (2004) [2]	Assumption of 0.02 utility decrement each day per month of heroin use	Assumption tested in sensitivity analyses only
Negrin (2006) [3]	NR	Nottingham Health Profile as reported in a multicentre study comparing three MMT programmes in five drug treatment centres in Barcelona
Schackman	Utility adjustment applied to all health	Based on utility weights from a societal perspective study

CONFIDENTIAL

(2012) [4]	states in the model for in treatment, off drugs; in treatment, on drugs, out of treatment, off drugs and out of treatment, on drugs	which used a panel of UK general population members to make valuations on given health states using standard gamble method
Stephen (2012) [5]	Utility adjustments used for 'Untreated or relapse, reduced use of heroin, and heroin-free states	Based on utility weights from a societal perspective study which used a panel of UK general population members to make valuations on given health states using standard gamble method
Tran (2012) [6]	Utility adjustment applied for health states: ART and on MMT ongoing drug abuse; ART and on MMT ongoing drug abstinence	Based on mapping of World Health Organisation Quality of life-Brief version (WHOQOL-BREF) to QALYs
Zaric (2000)	Utility based on infection status and IDU in MMT, IDU not in MMT and non	Assumptions based on literature on quality of life impacts in

CONFIDENTIAL

[7]	IDU	non-AIDS HIV and AIDS
Zaric (2000) [8]	Utility based on infection status and IDU in MMT, IDU not in MMT and non IDU	Assumptions based on literature on quality of life impacts in non-AIDS HIV and AIDS
Adi (2007) [9]	Mean utilities on NAL, placebo and not on treatment	Bespoke utility study based on utility weights from a societal perspective study which used a panel of UK general population members to make valuations on given health states using standard gamble method
Connock (2007) [10]	Mean utilities on MMT and BMT for first 2 weeks then week 3-52	Based on utility weights from a societal perspective study which used a panel of UK general population members to make valuations on given health states using standard gamble methods study
Schering-	NR	Full details not available, but based on published literature.

CONFIDENTIAL

Plough (2007)<br><br>[11]		

AIDS, acquired immunodeficiency syndrome; ART, ; BMT, buprenorphine maintenance treatment; HIV, human immunodeficiency virus; IDU, injecting drug user; MMT, methadone maintenance treatment; NAL, naltrexone; NR, not reported; QALY, quality-adjusted life-year; UK, United Kingdom.

References

1. Barnett PG, Zaric GS, Brandeau ML. The cost-effectiveness of buprenorphine maintenance therapy for opiate addiction in the United States. Addiction. 2001;96:1267-78.
2. Masson CL, Barnett PG, Sees KL, Delucchi KL, Rosen A, Wong W et al. Cost and cost-effectiveness of standard methadone maintenance treatment compared to enriched 180-day methadone detoxification. Addiction. 2004;99:718-26.
3. Negrin MA, Vazquez-Polo FJ. Bayesian cost-effectiveness analysis with two measures of effectiveness: the cost-effectiveness acceptability plane. Health Econ. 2006;15:363-72.

CONFIDENTIAL

4. Schackman BR, Leff JA, Polsky D, Moore BA, Fiellin DA. Cost-effectiveness of long-term outpatient buprenorphine-naloxone treatment for opioid dependence in primary care. J Gen Intern Med. 2012;27:669-76.

5. Stephen JH, Halpern CH, Barrios CJ, Balmuri U, Pisapia JM, Wolf JA et al. Deep brain stimulation compared with methadone maintenance for the treatment of heroin dependence: a threshold and cost-effectiveness analysis. Addiction. 2012;107:624-34.

6. Tran BX, Ohinmaa A, Duong AT, Nguyen LT, Vu PX, Mills S et al. The cost-effectiveness and budget impact of Vietnam's methadone maintenance treatment programme in HIV prevention and treatment among injection drug users. Glob Public Health. 2012;7:1080-94.

7. Zaric GS, Barnett PG, Brandeau ML. HIV transmission and the cost-effectiveness of methadone maintenance. Am J Public Health. 2000;90:1100-11.

8. Zaric GS, Brandeau ML, Barnett PG. Methadone maintenance and HIV prevention: A cost-effectiveness analysis. Manage Sci. 2000;46:1013-31.

9. Adi Y, Juarez-Garcia A, Wang D, Jowett S, Frew E, Day E et al. Oral naltrexone as a treatment for relapse prevention in formerly opioid-dependent drug users: a systematic review and economic evaluation. Health Technol Assess. 2007;11:iii-iv, 1-85.

CONFIDENTIAL

10. Connock M, Juarez-Garcia A, Jowett S, Frew E, Liu Z, Taylor RJ et al. Methadone and buprenorphine for the management of opioid dependence: a systematic review and economic evaluation. Health Technol Assess. 2007;11:1-171, iii-iv.

11. Schering-Plough. Manufacturer's submission. Cited in Connock et al. Health Technol Assess. 2007;11:1-171, iii-iv.

CONFIDENTIAL
