# Supplementary material for: A systematic review of health economic models of opioid agonist therapies in maintenance treatment of non-prescription opioid dependence
Source: Addict Sci Clin Pract. 2017 Feb 24;12:6. doi: 10.1186/s13722-017-0071-3 (PMC5324212; doi:10.1186/s13722-017-0071-3)
Supplement: Supplementary file 6 — Additional file 6. Summary of resource utilisation and costs. [file 13722_2017_71_MOESM6_ESM.pdf]

Additional File 6. Summary of resource utilisation and costs

Reference	Details	Source/Notes
Barnett (1999) [1]	MMT costs	Based on average cost of MMT in the US. Costs provided as summary estimate, no details on constituent components of this cost.
Barnett (2001) [2]	Drug costs	No costs were available at the time, so authors estimated plausible range for take home buprenorphine.
	Urinalysis, specialised dispensary costs, physician evaluations, psychological interventions	Based on published data on evaluation of methadone programmes by same authors
	HIV medication and treatment	NR
	Cost of treatment of co-morbid illnesses	NR
Masson (2004) [3]	Methadone treatment costs including counselling, education, ongoing medical care, dispensing	Resource use based on microcosting from an RCT comparing MMT to 180-day psychosocially enriched

CONFIDENTIAL

	costs and urine collection costs	detoxification for treatment of opioid dependence, the NTORS study - the largest UK drug treatment outcome study at the time (1995-2000), and local cost data for labour and wages
	Capital and fixed costs including staff costs and facility costs	
	Healthcare utilisation including hospital stays, emergency room visits, ambulatory medical care	
	Substance abuse and mental health treatment costs	
Schackman (2012) [4]	Medication costs	Resource use and cost data obtained from same prospective observational study providing treatment retention rates and combination of published cost studies, and published unit costs.
	Provider costs including physician time, nursing time, overhead, laboratory testing)	
	Patient costs (travel time, visit time, transport)	
Sheerin (2004) [5]	Methadone treatment costs including assessment and basic counselling	Based on costs of a methadone treatment centre

CONFIDENTIAL

	Operating costs of MMT (staff, facilities, lab testing)	
	Cost of HCV treatment	Based on data sourced from Australian Pharmaceutical Benefit Schedule
Stephen (2012) [6]	MMT treatment	Based on published source describing the cost of concordance with opiate substitution treatment guidelines.
	Societal costs of continued drug addiction including medical care, cost of complications and co-morbidities, support services, medical care for crime victims, productivity losses of victims and addicts, crime costs	Combination of publicly available reference sources and healthcare databases.
	DBS costs	Combination of publicly available reference sources and healthcare databases.
	DBS complications	
Tran (2012)	ART drug costs	Vietnam Ministry of Health costing study of ART and

CONFIDENTIAL

[7]		data sourced from the longitudinal cohort study across 6 MMT clinics in Vietnam
	Variable costs such as staff, MMT, urine tests and consumables)	
	Fixed costs such as facility costs, operating costs)	
Zaric (2000) [8]	HIV care	US statistical data and published literature
	Non-HIV related costs	
	MMT	
Zaric (2000) [9]	MMT	Based on published literature and administration databases
	Non-HIV related costs	
	HIV care costs	
Zarkin (2005) [10]	MMT treatment	Based on previous publication by same authors

CONFIDENTIAL

	Costs associated with crime	Published literature
	Productivity costs based on individual earnings	Published literature
	Healthcare costs including inpatient, outpatient and emergency costs	Published literature
Miller (2004) [11]	Hospitalisation and emergency use costs	Open cohort study and HIV Drug treatment programme
	Costs and resources associated with crime	Data from police force, open cohort study and public cost data
	Employment rates	Open cohort study
Adi (2007) [12]	NAL treatment costs including counselling and urine tests	Based on a randomized double-blind trial with 405 opioid-dependent patients in which buprenorphine was compared to methadone maintenance therapy
	Healthcare services, the CJS and employment	NTORS study - largest prospective longitudinal cohort study of treatment outcome for drug misusers ever conducted in the UK
	Healthcare resources such as GP visits, inpatient	

CONFIDENTIAL

	hospital stays, mental health visits and A&E	
Connock (2007) [13]	BMT and MMT treatment costs including counselling and urine tests	Based on a randomized double-blind trial with 405 opioid-dependent patients in which buprenorphine was compared to methadone maintenance therapy
	Healthcare services, the CJS and employment	NTORS study - largest prospective longitudinal cohort study of treatment outcome for drug misusers ever conducted in the UK
	Healthcare resources such as GP visits, inpatient hospital stays, mental health visits and A&E	
Schering-Plough (2007) [14]	BMT and MMT treatment costs including counselling and urine tests	Based on a randomized double-blind trial with 405 opioid-dependent patients in which buprenorphine was compared to methadone maintenance therapy
	Time to dispense and supervise methadone and buprenorphine	Time in motion study
	Health care resource usage	NTORS study - largest prospective longitudinal cohort study of treatment outcome for drug misusers ever conducted in the UK

CONFIDENTIAL

SMC (2007) [15]	Costs of BMT/NAL, BMT and MMT (oral and injection)	Data were taken from company SMC submission, NICE submission and SPC

A&E, accident and emergency; ART, anti-retroviral treatment; BMT, buprenorphine maintenance treatment; CJS, criminal justice system; DBS, deep brain stimulation; GP, general practitioner; HCV, hepatitis C virus; MMT, methadone maintenance treatment; NAL, naltrexone; NICE, National Institute for Health and Care Excellence; NR, not reported; NTORS, National Treatment Outcome Research Study; RCT, randomised controlled trial; SMC, Scottish Medicines Consortium; SPC, summary of product characteristics; UK, United Kingdom.

References

CONFIDENTIAL

1. Barnett PG. The cost-effectiveness of methadone maintenance as a health care intervention. Addiction. 1999;94:479-88.
2. Barnett PG, Zaric GS, Brandeau ML. The cost-effectiveness of buprenorphine maintenance therapy for opiate addiction in the United States. Addiction. 2001;96:1267-78.
3. Masson CL, Barnett PG, Sees KL, Delucchi KL, Rosen A, Wong W et al. Cost and cost-effectiveness of standard methadone maintenance treatment compared to enriched 180-day methadone detoxification. Addiction. 2004;99:718-26.
4. Schackman BR, Leff JA, Polsky D, Moore BA, Fiellin DA. Cost-effectiveness of long-term outpatient buprenorphine-naloxone treatment for opioid dependence in primary care. J Gen Intern Med. 2012;27:669-76.
5. Sheerin IG, Green FT, Sellman JD. What is the cost-effectiveness of hepatitis C treatment for injecting drug users on methadone maintenance in New Zealand? Drug Alcohol Rev. 2004;23:261-72.
6. Stephen JH, Halpern CH, Barrios CJ, Balmuri U, Pisapia JM, Wolf JA et al. Deep brain stimulation compared with methadone maintenance for the treatment of heroin dependence: a threshold and cost-effectiveness analysis. Addiction. 2012;107:624-34.
7. Tran BX, Ohinmaa A, Duong AT, Nguyen LT, Vu PX, Mills S et al. The cost-effectiveness and budget impact of Vietnam's methadone maintenance treatment programme in HIV prevention and treatment among injection drug users. Glob Public Health. 2012;7:1080-94.
8. Zaric GS, Barnett PG, Brandeau ML. HIV transmission and the cost-effectiveness of methadone maintenance. Am J Public Health. 2000;90:1100-11.
9. Zaric GS, Brandeau ML, Barnett PG. Methadone maintenance and HIV prevention: A cost-effectiveness analysis. Manage Sci. 2000;46:1013-31.
10. Zarkin GA, Dunlap LJ, Hicks KA, Mamo D. Benefits and costs of methadone treatment: results from a lifetime simulation model. Health Econ. 2005;14:1133-50.
11. Miller CL, Schechter MT, Wood E, Spittal PM, Li K, Laliberte N et al. The potential health and economic impact of implementing a medically prescribed heroin program among Canadian injection drug users. Int J Drug Policy. 2004;15:259-63.
12. Adi Y, Juarez-Garcia A, Wang D, Jowett S, Frew E, Day E et al. Oral naltrexone as a treatment for relapse prevention in formerly opioid-dependent drug users: a systematic review and economic evaluation. Health Technol Assess. 2007;11:iii-iv, 1-85.
13. Connock M, Juarez-Garcia A, Jowett S, Frew E, Liu Z, Taylor RJ et al. Methadone and buprenorphine for the management of opioid dependence: a systematic review and economic evaluation. Health Technol Assess. 2007;11:1-171, iii-iv.
14. Schering-Plough. Manufacturer's submission. Cited in Connock et al. Health Technol Assess. 2007;11:1-171, iii-iv.
15. Scottish Medicines Consortium. Buprenorphine/naloxone 2mg/0.5mg, 8/2mg sublingual tablet (Suboxone). No. 355/07. 2007. https://www.scottishmedicines.org.uk/files/buprenorphine_naloxone_sublingual_tablet__Suboxone__355-07_.pdf. Accessed 3 Jun 2015.
CONFIDENTIAL
